# Adrenalectomy for a Large Non‐Functional Adrenocortical Tumor With Aggressive Histopathologic Features: A Clinical Case Report

**DOI:** 10.1002/ccr3.73740

**Published:** 2026-10-23

**Authors:** Jawad Khan, Abdul Eizad Asif, Hammad Yousaf, Haider Ali Nawaz, Muhammad Hassaan, Samiya Rahim, Farzana Yeasmin, Muddassir Khalid, Fred Segawa

**Affiliations:** ^1^ Department of Urology Ayub Teaching Hospital Abbottabad Khyber Pakhtunkhwa Pakistan; ^2^ Department of Internal Medicine Shalamar Medical and Dental College Lahore Punjab Pakistan; ^3^ Department of Internal Medicine Allama Iqbal Medical College Lahore Punjab Pakistan; ^4^ Shaheed Suhrawardy Medical College Dhaka Bangladesh; ^5^ Department of Medicine Nishtar Medical University Multan Punjab Pakistan; ^6^ Department of Medicine Makerere University College of Health Sciences Kampala Central Region Uganda

**Keywords:** adrenalectomy, adrenocortical carcinoma, case report, open surgery, surgical oncology, urology

## Abstract

Adrenocortical tumors are infrequent neoplasms, ranging from benign adenomas to aggressive carcinomas. They usually have an unspecific clinical presentation, thus being a challenge in diagnosis and treatment. The following case report discusses the management of a large, non‐functional adrenocortical tumor with histologically aggressive features, underlining the diagnostic workup, the multidisciplinary surgical approach, and the short‐term postoperative outcome after adrenalectomy. We report the case of a 40‐year‐old female patient presenting with dull left flank pain and a large, non‐functional adrenal mass. She underwent open left adrenalectomy. The resected specimen measured 8.4 × 7.6 × 6.5 cm and was histopathologically diagnosed as adrenocortical carcinoma. She was discharged on the 4th post‐operative day and at the 3‐month follow‐up was found to be disease‐free on imaging.

## Key Clinical Message

Large, non‐functional adrenal masses necessitate a high index of suspicion for adrenocortical carcinoma. A comprehensive multidisciplinary approach, including hormonal profiling and oncologically sound surgical resection, is vital. Despite aggressive histopathology, early radical adrenalectomy remains the cornerstone for achieving a disease‐free status and improving survival.

## 1 Background

Adrenocortical tumors encompass a broad spectrum of neoplastic conditions, ranging from benign adenomas to highly aggressive carcinomas. ACC is one of the rarest malignancies, comprising less than 0.2% of all malignancies, with an annual incidence of 0.7–2 per million [1, 2]. Many of these present incidentally on imaging for unrelated causes due to their vague or absent symptoms. Management of ACC is a real challenge because of its rarity and inhomogeneity in presentation and limited systemic therapies. Resection remains the only curative approach in localized disease [3, 4]. Among the radiologic findings, tumor size > 6 cm, heterogeneous enhancement, and necrosis raise suspicion for malignancy, whereas histopathologic scoring systems confirm diagnosis and guide prognosis, including the Modified Weiss or Helsinki systems [5, 6, 7]. This case report has been prepared in line with the CARE checklist 2025 guidelines.

## 2 Case History

A 40‐year‐old female presented to the Urology outpatient department with a 3‐month history of dull, non‐radiating left flank pain. There were no constitutional symptoms, hematuria, hypertension, palpitations, or weight changes. Physical examination revealed mild tenderness in the left flank region, with no palpable mass or additional abnormalities.

An initial ultrasound abdomen and pelvis showed a heterogeneous left suprarenal mass, measuring approximately 7.6 × 5.1 cm, hypoechoic with mixed echogenicity, arising from the upper pole of the left kidney. No hydronephrosis or renal calculi were noted (Figure 1).

**FIGURE 1:**
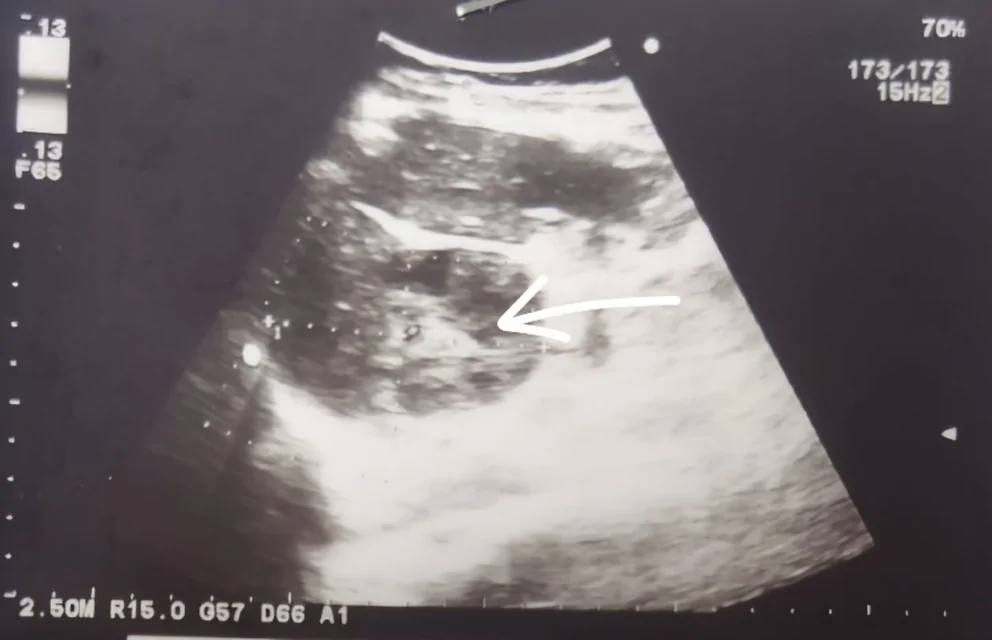
Transverse ultrasound image demonstrating a well‐defined hypoechoic nodule (indicated by an arrow/marker) in the left adrenal gland, measuring approximately 7.6 × 5.1 cm.

Based on the clinical presentation of a large (8.4 cm), non‐functional left adrenal mass in a 40‐year‐old patient, the differential diagnosis must account for both primary adrenal pathologies and secondary lesions.

## 3 Differential Diagnosis

1. Adrenocortical carcinoma (ACC): The primary suspicion for any large (> 4–6 cm), unilateral adrenal mass. While many are functional, non‐functional variants often present late with local symptoms like flank pain.
2. Adrenocortical adenoma (non‐functioning): Though typically smaller than 4 cm and characterized by high lipid content on imaging, large macroadenomas can occur. However, aggressive histopathologic features rule this out postoperatively.
3. Pheochromocytoma: Often referred to as the 10% tumor. Even without classic hypertensive paroxysms, it must be excluded via plasma or urinary metanephrines before surgery to prevent intraoperative crisis.
4. Adrenal metastasis: The adrenal glands are common sites for secondary spread from primary tumors in the lung, breast, kidney, or melanoma.
5. Adrenal myelolipoma: A benign tumor composed of mature adipose tissue and hematopoietic elements. These can grow quite large but usually show characteristic fat density on CT.
6. Ganglioneuroma: A rare, benign tumor originating from neural crest cells. They are typically slow‐growing, non‐functional, and can reach significant sizes in younger adults.

### 3.1 Investigations

A contrast‐enhanced CT scan of the abdomen and pelvis demonstrated a well‐defined heterogeneous left adrenal mass, measuring 8.4 × 7.6 × 6.5 cm, showing heterogeneous post‐contrast enhancement with necrotic areas (Figures 2 and 3).

**FIGURE 2:**
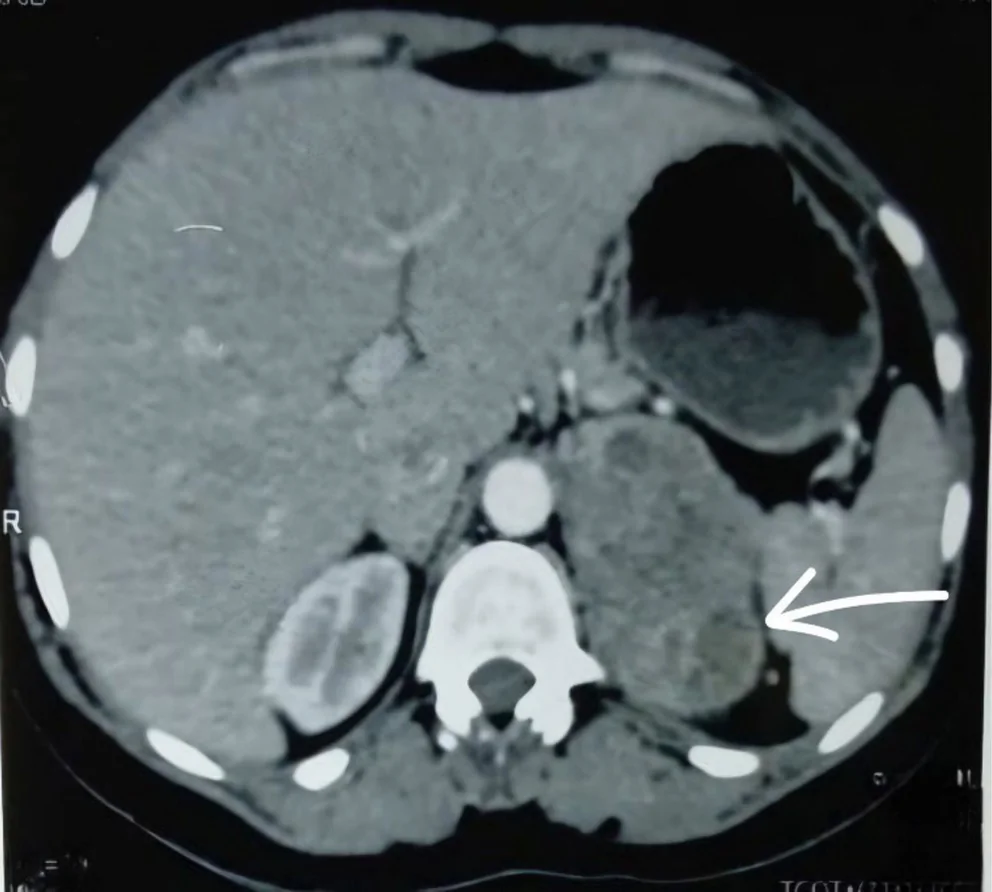
Axial view of the contrast‐enhanced CT abdomen showing a well defined heterogeneous mass (arrow) in the left adrenal gland, measuring approximately 8.0 × 6.5 cm.

**FIGURE 3:**
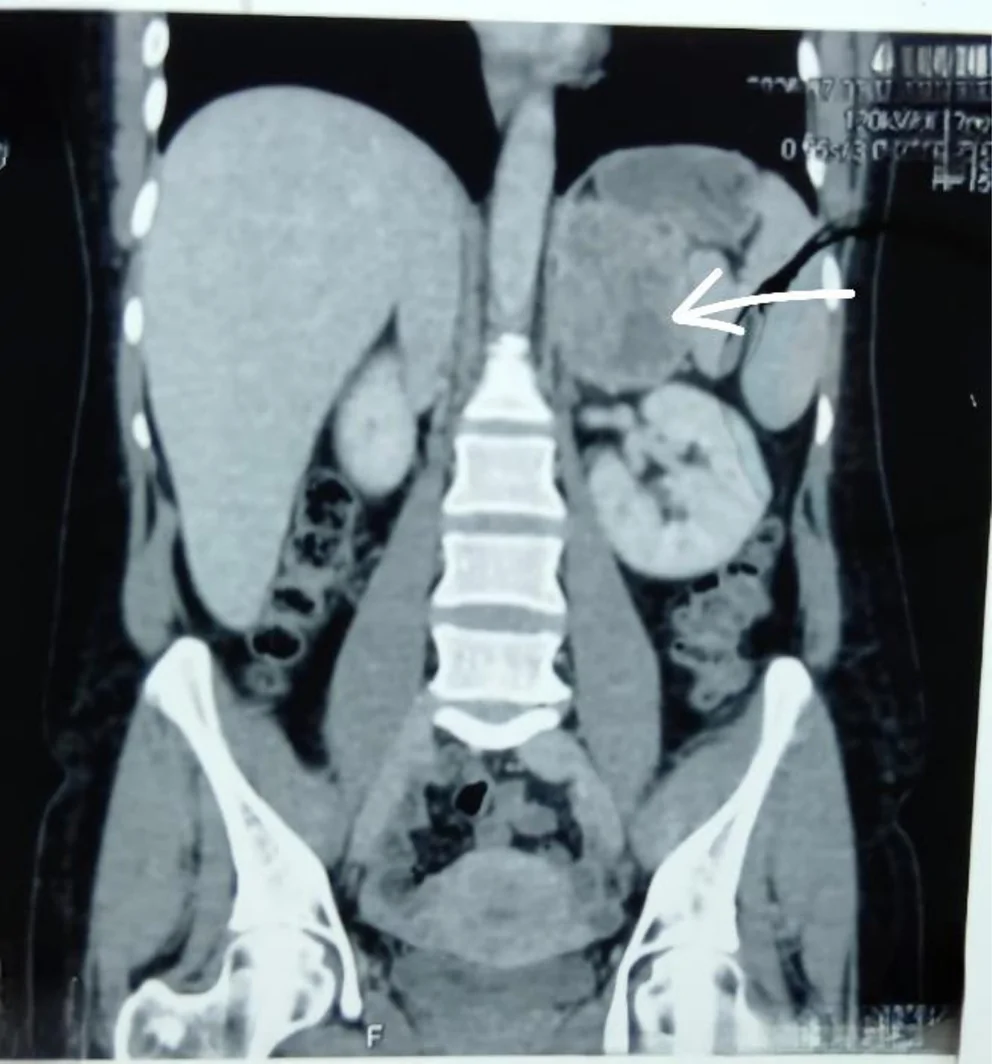
Coronal view of the contrast‐enhanced CT abdomen confirming the presence of the left adrenal nodular mass (arrow) located above the left kidney.

The mass abutted the spleen, tail of pancreas, upper pole of left kidney, and the left diaphragmatic crus, without definite invasion of adjacent organs. No calcification, regional lymphadenopathy, or distant lesions were identified.

A complete functional adrenal evaluation was performed, including serum metanephrines, normetanephrines, 24‐h urinary cortisol, and aldosterone‐renin ratio; all values were within normal limits (Table 1).

**TABLE 1:** Summary of relevant lab investigations.

Lab test	Result value	Normal reference range
Urine free cortisol	23.1	4.6 − 28.6 μg/day
Serum creatinine	0.8	0.5 −1.1mg/dL
Normetanephrine free (plasma)	128.46	0 −190pg/mL
Norepinephrine free (plasma)	38.84	0 −50pg/mL
Urine metanephrine (24 h)	64.32	Less than 350 μ g/day
Serum aldosterone	13.57	3.3 −23.3ng/dL
Aldosterone/Direct renin ratio	9.5	

Baseline laboratory investigations were also unremarkable. Following multidisciplinary team (MDT) discussion, the patient was optimized for surgery and subsequently underwent a left open adrenalectomy under general anesthesia via a left hemichevron incision in the supine position. The surgery was uneventful, and the mass was excised completely (Figure 4).

**FIGURE 4:**
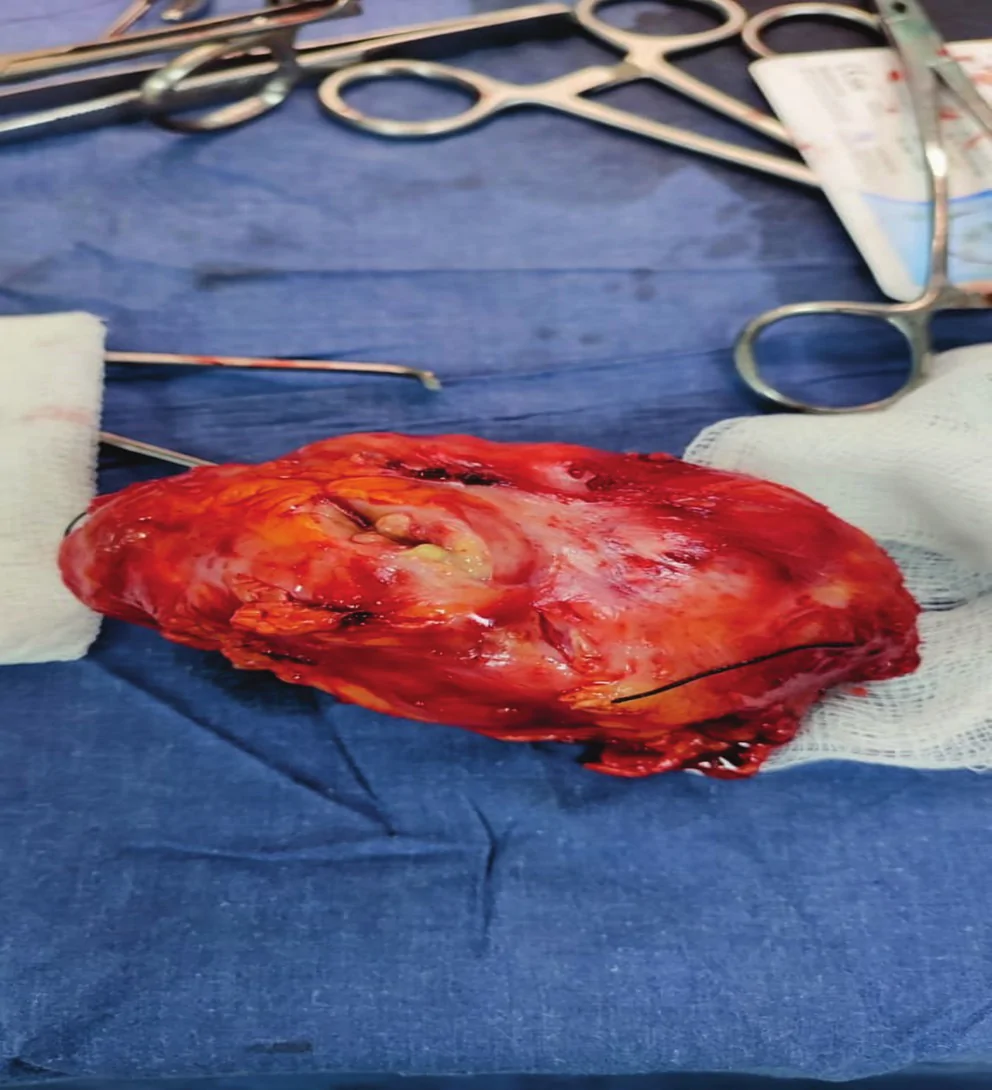
Gross specimen of the resected left adrenocortical tumor.

### 3.2 Management

Histopathology revealed an adrenocortical neoplasm composed of pleomorphic polygonal cells with eosinophilic to clear cytoplasm. There was mitotic activity > 5/50 HPF, atypical mitoses, and tumor necrosis (Figure 5). Lymphovascular invasion was identified, and the tumor was seen invading periadrenal adipose tissue, consistent with pT3 disease. Margins were free, with the lesion 0.1 cm from the inked margin. The Modified Weiss score was 6, indicating malignant behavior.

**FIGURE 5:**
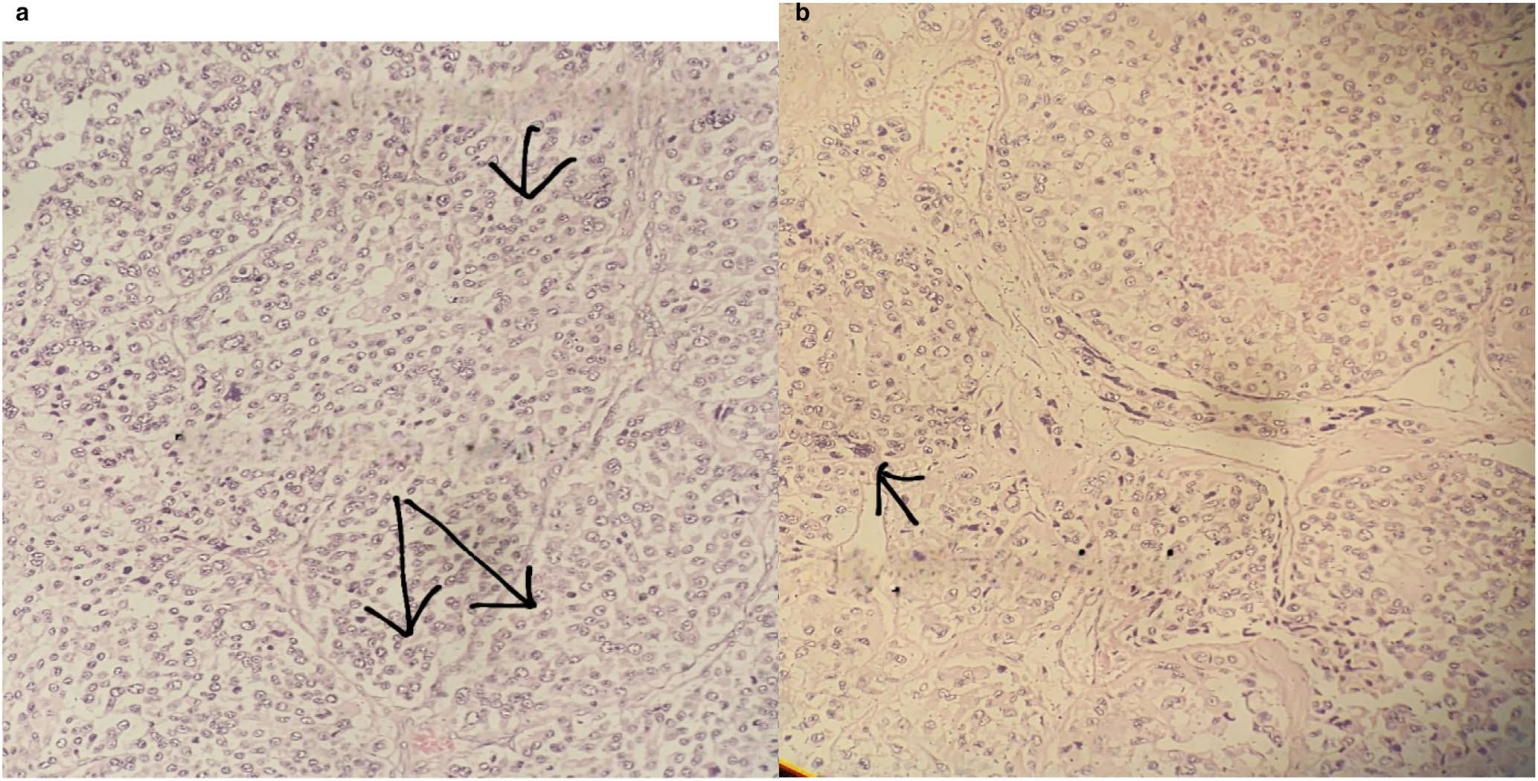
(a) Histopathological features of adrenocortical carcinoma (Weiss score 6). (a) Section showing marked cellular pleomorphism and a trabecular growth pattern (H&E stain). (b) High‐power view demonstrating an atypical mitotic figure (arrow), contributing to the high mitotic count and malignant Weiss score (H&E stain).

### 3.3 Outcomes and Follow‐Up

Postoperatively, the patient was reviewed again in the Urology MDT, where a consensus was reached to maintain strict surveillance with regular follow‐up due to the malignant potential of the tumor. The patient's first follow‐up at 3 months included relevant laboratory tests and a CT scan of the abdomen and pelvis, which showed no evidence of local recurrence or systemic metastasis.

## 4 Discussion

ACC is a rare endocrine malignancy with variable biological behavior and poor long‐term prognosis [1, 3, 8]. The majority of patients present with large, non‐functional masses, often greater than 6 cm, and are diagnosed incidentally [9]. Radiologic features concerning malignancy include tumor heterogeneity, necrosis, and irregular borders [10]. Surgical excision remains the cornerstone of curative therapy. In large or potentially invasive tumors, open adrenalectomy offers improved margin control and reduces the risk of intra‐operative tumor spillage [11, 12, 13]. In this case, the open approach allowed a complete removal with negative margins and minimal morbidity. Histopathologic assessment based on the Modified Weiss criteria confirmed the diagnosis, with a score of 6 suggesting malignant potential [5]. A study about diagnostic and prognostic assessments of adrenocortical carcinomas by pathological features, immunohistochemical markers and reticular histochemical staining showed six immunohistochemical antibodies (MelanA, Ki‐67, IGF‐2, β‐catenin, P53 and CYP11B1) and reticulin framework alterations showed diagnostic value. Notably, Ki‐67 and reticulin staining were most recommended. Evident reticulin staining was frequently present in ACCs (*p* < 0.001). Ki‐67 was significantly higher in ACCs (*p* < 0.001) [14]. Multidisciplinary discussion optimized preoperative planning and postoperative follow‐up, in keeping with best‐practice guidelines [2, 15]. This case demonstrates that early identification and multidisciplinary care can significantly improve short‐term outcomes, even in large tumors. Long‐term follow‐up will be necessary given the recurrence rates of up to 70% within 2 years [16]. Current treatment guidelines recommend adjuvant mitotane after resection of adrenocortical carcinoma with high‐risk features (e.g., tumor rupture, positive margins, positive lymph nodes, high grade, elevated mitotic index, and advanced stage) however a study conducted on the Outcomes of Adjuvant Mitotane after Resection of Adrenocortical Carcinoma: A 13‐Institution Study by the US Adrenocortical Carcinoma Group suggest that adjuvant mitotane after resection of adrenocortical carcinoma is not associated with improved recurrence‐free survival and overall survival [17].

## 5 Conclusion

Large non‐functional adrenal masses suspicious for malignancy should be promptly treated with surgical resection. To this date, open adrenalectomy remains standard for achieving negative margins and optimal oncologic outcomes. This case emphasizes the importance of multidisciplinary evaluation, accurate histopathological assessment, and organized postoperative surveillance.

## Author Contributions

**Jawad Khan:** conceptualization, supervision. **Abdul Eizad Asif:** data curation, methodology. **Hammad Yousaf:** visualization, investigation. **Haider Ali Nawaz:** writing – original draft. **Muhammad Hassaan:** writing – original draft. **Samiya Rahim:** resources. **Farzana Yeasmin:** writing – original draft. **Muddassir Khalid:** writing – review and editing, project administration, validation. **Fred Segawa:** resources.

## Funding

The authors have nothing to report. All authors certify that they have no affiliation with or involvement in any organization or entity with any financial interest or non‐financial interest in the subject matter or materials discussed in this manuscript.

## Ethics Statement

The authors have nothing to report.

## Consent

Written informed consent was obtained from the patient for publication of this case report and accompanying images. A copy of the written consent is available for review by the Editor‐in‐Chief of this journal on request.

## Conflicts of Interest

The authors declare no conflicts of interest.

## Data Availability

Data available on request from the authors.
